# Association of caspase 8 polymorphisms -652 6N InsDel and Asp302His with progression-free survival and tumor infiltrating lymphocytes in early breast cancer

**DOI:** 10.1038/s41598-019-47601-x

**Published:** 2019-08-29

**Authors:** Jan Dominik Kuhlmann, Hagen Sjard Bachmann, Theresa Link, Pauline Wimberger, Eric Kröber, Christoph Thomssen, Brahima Mallé, Daniel Bethmann, Martina Vetter, Eva Johanna Kantelhardt

**Affiliations:** 1 Department of Gynecology and Obstetrics, Medical Faculty and University Hospital Carl Gustav Carus, Technische Universität Dresden, Dresden, Germany; 2 National Center for Tumor Diseases (NCT), Dresden, Germany: German Cancer Research Center (DKFZ), Heidelberg, Germany; Faculty of Medicine and University Hospital Carl Gustav Carus, Technische Universität Dresden, Dresden, Germany; Helmholtz-Zentrum Dresden-Rossendorf (HZDR), Dresden, Germany; 3 German Cancer Consortium (DKTK), Dresden and German Cancer Research Center (DKFZ), Heidelberg, Germany; 4 Institute of Pharmacology and Toxicology, Centre for Biomedical Education and Research (ZBAF), Witten/Herdecke University, Witten, Germany; 5 Institute of Pharmacogenetics, University Hospital Essen, University of Duisburg-Essen, Essen, Germany; 6 Department of Gynecology, Martin-Luther-University Halle-Wittenberg, Halle (Saale), Germany; 7 Institute of Pathology, Martin-Luther-University Halle-Wittenberg, Halle (Saale), Germany; 8 Insitute of Medical Epidemiology, Bioinformatics and Statistics, Martin-Luther-University Halle-Wittenberg, Halle (Saale), Germany

**Keywords:** Cancer genetics, Breast cancer

## Abstract

The caspase 8 variants *CASP8* -652 6N InsDel and Asp302His have previously been identified to promote survival of T-lymphocytes and to indicate reduced breast cancer susceptibility. Besides some preliminary findings, prognostic relevance of these polymorphisms in patients with existing breast cancer has not been investigated. Considering an immunomodulatory role of these polymorphisms, we genotyped 785 early breast cancer patients and correlated caspase 8 variants with disease-free survival (DFS) and the presence of tumor infiltrating lymphocytes (TILs). Early breast cancer specimens were collected as part of the multicenter prospective PiA study. Genotyping was performed by pyrosequencing, TILs status was assessed using hematoxylin & eosin staining. The *CASP8* -652Del variant was significantly associated with improved DFS in an allele-dose dependent manner (p = 0.027). Homozygosity for the -652Del variant was an independent predictor for improved DFS (HR = 0.36; 95% CI = 0.174–0.726; p = 0.005). In patients with the 302HisHis genotype, there was no event of recurrence during observation time. Combined analysis of diplotypes revealed an influence of both polymorphisms on DFS (p = 0.029). Interestingly, patients with the 302HisHis variant among the unstratified patient cohort (and among the luminal-like subtype, by trend) had tumors with lower lymphocyte infiltration (p = 0.025). We propose a prognostically favorable role of the -652Del and the 302His variant in primary breast cancer and suggest for the first time an association between polymorphisms in apoptosis-related genes and the immunophenotype in breast cancer. Our findings encourage further investigation of caspase 8 polymorphisms as biomarkers for prognostic and immunotherapeutic considerations.

## Introduction

Programmed cell death, also known as apoptosis, is an important process in multicellular organisms and is dysregulated during malignant progression. Caspase 8 is a 55 kDa cysteine protease. It contributes to intrinsic and extrinsic apoptosis initiation among the death receptor pathway and is regulated by additional upstream signalling pathways, such as the cathepsin/urokinase-type plasminogen activator receptor (uPAR) axis^1–3^. A decade ago, the *CASP8* polymorphisms, *CASP8* -652 AGTAAG InsDel (-652 6N InsDel, rs3834129) and *CASP8* Asp302His (rs1045485) were first described^4,5^. The non-coding *CASP8* -652 6N InsDel polymorphism describes a 6-bp deletion in the promoter region of the *CASP8* gene and parallels altered *CASP8* mRNA expression. It was reported that this deletion disrupts the binding site of the Specificity Protein 1 (Sp1) transcription factor within the caspase 8 promoter, leading to decreased caspase 8 transcription and decreased total caspase 8 activity. Considering the involvement of caspase 8 in apoptosis induction of lymphocytes, the *CASP8* -652 InsDel polymorphism was reported to promote survival of T-lymphocytes *in vitro*. In particular, a key publication reported that the *CASP8* -652 deletion allele is associated with impaired caspase 8 activity in T-lymphocytes and consequently with lower “*activation induced cell death*” (AICD) after exposure to cancer cell antigens^4^. Based on the assumption that this polymorphism has an immunomodulatory role, it was further reported that the *CASP8* -652Del variant significantly decreases breast cancer susceptibility in healthy individuals. This was explained by the fact that -652Del reduces the apoptotic threshold of lymphocytes, which are responsible for “tumor surveillance”, i.e. the detection and elimination of pre-malignant lesions^6^. Consequently, this is supposed to decrease the overall probability of breast cancer onset during lifetime^4^. The other polymorphism, namely *CASP8* Asp302His, is part of the coding region of caspase 8, causes an aspartic acid to histidine substitution and was likewise associated with decreased breast cancer susceptibility^4^. Although instructive experimental data on the functionality of *CASP8* Asp302His is missing, it has been speculated that the Asp302His substitution could as well decrease caspase 8 function^7^.

Besides the involvement of *CASP8* -652 InsDel and *CASP8* Asp302His in cancer susceptibility, recent studies investigated the possible impact of both polymorphisms on the outcome of patients with existing cancer. The -652Del allele or the 302His allele were associated with poor survival in colon cancer patients or neuroblastoma patients, respectively^8,9^. However, prognostic relevance of both caspase 8 alleles for breast cancer patients remains to be determined. Furthermore, considering an immunomodulatory capacity of these polymorphisms, it is completely unknown, whether these variants may influence tumor immune infiltration, reflected by tumor infiltrating lymphocytes (TILs). In breast cancer patients, clinical relevance of TILs has extensively been studied^10,11^. It is known, for instance, that increased TIL concentration predicts response to neoadjuvant chemotherapy across all intrinsic subtypes and is associated with improved survival in HER2-positive and triple negative breast cancer (TNBC)^12^. Moreover, defined TIL subpopulations, such as CD8^+^ T-lymphocytes, have already been discussed as potential response predictors for immunotherapeutic drugs, such as checkpoint inhibitors^13^.

We assume that functional polymorphisms, which are associated with cancer susceptibility, are also likely to influence prognosis of an existing cancer. Therefore, the objective of the current study was to analyze 785 clinically documented breast cancer patients for the *CASP8* -652 6N Ins/Del and *CASP8* Asp302His polymorphism and to correlate resulting genotypes with disease-free survival (DFS) and the presence of TILs.

## Methods

All methods were performed in accordance with the relevant guidelines and regulations.

### Patient characteristics

Fresh frozen primary tumor specimens (n = 785) from women with a histologically confirmed primary diagnosis of invasive carcinoma of the breast (International Classification of Disease-Oncology [ICD-O-3] codes C50.0–9) without evidence of distant metastasis were collected as part of the multicenter prospective PiA cohort (NCT 01592825) at the Martin-Luther-University Halle-Wittenberg between 2009 and 2011. The study was approved by the ethics committee of the Martin-Luther University Halle-Wittenberg 15.09.2009 and 10.03.2010 for patient recruitment, (15.09.2016 for this sub-protocol) and informed consent had been obtained from each patient. Tumor specimens were fresh frozen after primary surgery and stored at −80 °C until further use. Clinicopathological parameters were obtained for each patient and documented using SPSS 24 (SPSS Inc., Chicago, Illinois, USA). TNM-staging system was used^14^. Patient information was pseudo-anonymized prior to analysis. Receptor defined breast cancer subtypes were determined according to the St. Gallen classification^15^. Due to missing Ki-67 values, we used histopathological grading to assess cell proliferation^16^.

### DNA extraction and caspase 8 genotyping

Genomic DNA of fresh frozen breast cancer tissue was purified with the QIAamp DNA Mini Kit (Qiagen, Hilden, Germany). *CASP8* -652 6N InsDel and *CASP8* Asp203His genotypes were determined by pyrosequencing (Biotage, Uppsala, Sweden), according to the manufacturer’s instructions. First, the genomic caspase 8 regions of interest were amplified using the “slowdown” polymerase chain reaction (PCR)^17^, with the following primer sequences: -652 6N forward: 5′ BIOTIN-AACTTGCCCAAGGTCACG 3′, -652 6N reverse: 5′ TGAGGTCCCCGCTGTTAA 3′, 302 forward: 5′ GACCACGACCTTTGAAGAGCT 3′, and 302 reverse: 5′ BIOTIN-AGATTTGCTCTACTGTGCAGTCA 3′. PCR products were analyzed by pyrosequencing using sequencing primers -652 6N 5′ GTAATTCTTGCTCTGCC 3′ and 302 5′ TGAGATCAAGCCCCA 3′ on the PSQ96 system, according to the manufacturer’s instructions (Biotage, Uppsala, Sweden). Results were analyzed using the proprietary PSQ96 SNP software. Re-genotyping of 50 randomly selected samples to control for genotype failures revealed 100% concordance with the previously obtained results. All experimental methods have been performed, according to the guidelines, proposed by the Journal *Scientific Reports*.

Since blood-leucocytes were not available for most of the patients, we had to perform genotyping in early breast cancer tissue. However, in a parallel experiment, we performed genotyping of DNA of 45 breast cancer tissues and 45 corresponding blood-leucocytes. Since resulting genotypes were identical in all cases, we conclude that our analysis in the early tumor tissue describes constitutive changes in the germline of each patient and does not refer to somatic tumor-associated aberrations.

### Detection and quantification of tumor infiltrating lymphocytes

Tumor infiltrating lymphocytes were assessed scoring the hot spots of TILs modified according to “International TILs Working Group 2014”^18^. Intratumoral TILs were defined as intraepithelial mononuclear cells found within tumor cell nests or in direct contact with tumor cells. Stromal TILs were defined as the percentage of tumor stromal area containing a lymphocytic infiltrate with no direct contact with tumor cells. The percentage of TILs as semi-quantitative parameter was used: 0–10% = low; 20–40% = moderate; 50–90% = high.

### Statistical analysis

The standardized definitions for efficacy end points (STEEP) criteria were used as endpoint definitions^19^. The primary endpoint of this study was invasive DFS. Survival time equaled the time from the date of diagnosis to the date of event or to the date of last contact. Women without event were right-censored at the date of last contact. Information on survival was obtained in 2017. Statistical analyses were performed using GraphPad Prism 6.0 (GraphPad Software, LaJolla, CA, USA) and SPSS software version 24.0 (IBM, Armonk, NY, USA). Clinical variables and genotypes were compared using either Student’s t test, ANOVA for continuous variables or Pearson’s Chi² test for categorical data. Control for deviation from the Hardy–Weinberg equilibrium was conducted using a publically available Hardy–Weinberg equilibrium calculator^20^. Linkage disequilibrium and haplotypes were assessed using Haploview^21^. Kaplan-Meier plots and the log-rank test for trend were used to retrospectively evaluate the relationship between *CASP8* Asp302His genotypes, *CASP8* -652 6N InsDel genotypes, *CASP8* diplotypes, and outcome between the date of primary diagnosis and the end of follow-up. Both univariate analysis and stepwise backward multivariable Cox regression analysis were used to analyze the effect of genotypes and diplotypes of the *CASP8* polymorphisms on clinical outcome. Hazard ratios (HR) and 95% confidence intervals (95% CI) were calculated based on the Cox regression model. Differences with p-values < 0.05 were considered significant; all p-values are two-tailed. All experimental methods have been performed, according to the guidelines, proposed by the Journal *Scientific Reports*.

### Ethics approval and consent to participate

The study was approved by the ethics committee of the Martin-Luther University Halle-Wittenberg 15.09.2009 and 10.3.2010 for patient recruitment, (15.09.2016 for this sub-protocol) and informed consent had been obtained from each patient.

### Statement of translational relevance

The minor alleles of two caspase 8 polymorphisms, namely *CASP8* -652 6N InsDel and Asp302His, were shown to promote survival of T-lymphocytes and were associated with reduced breast cancer susceptibility. We genotyped 785 breast cancer patients and correlated caspase 8 variants with disease-free survival (DFS) and the presence of tumor infiltrating lymphocytes (TILs). We demonstrate a prognostically favorable role of -652Del and 302His in breast cancer and show for the first time an association between caspase 8 polymorphisms and the immunophenotype in breast cancer. Our findings add a new layer of complexity to immunological interaction in breast cancer and indicate that the immunophenotype of a given tumor could pre-defined by genetic variability in apoptosis regulating genes. This could be in addition to known factors affecting the immunophenotype, such as the rate of somatic mutations or the degree of neo-antigenicity.

## Results

### Prognostic relevance of CASP8 -652 6N InsDel in breast cancer patients

In 216/785 patients (27.5%), homozygosity for the deletion variant (DelDel) was observed, whereas 381/785 patients (48.5%) were heterozygous (InsDel) and 188/785 patients (23.9%) showed an Ins/Ins genotype (Supplementary Table 1). No significant deviation from Hardy-Weinberg equilibrium was detectable (p = 0.432) and the observed genotype distribution as well as the allelic frequencies (f_Ins_ = 0.482) were comparable to those previously reported in cancer cases and healthy controls of European ancestry^22^. Subsequently, we correlated *CASP8* -652 InsDel genotypes with the patients’ clinicopathological data, including hormone receptor and HER2-receptor status and the intrinsic breast cancer subtype (Supplementary Table 1). There was a significant correlation between the -652 6N InsDel polymorphism and HER2-receptor status (p = 0.026). Hence, the HER2-positivty rate in patients with the -652 6N DelDel variant was slightly higher than in patients with a -652 InsDel or -652 DelDel genotype. Apart from this association, -652 InsDel genotypes correlated neither with the intrinsic breast cancer subtypes, nor with other clinicopathological parameters.

**Figure 1:**
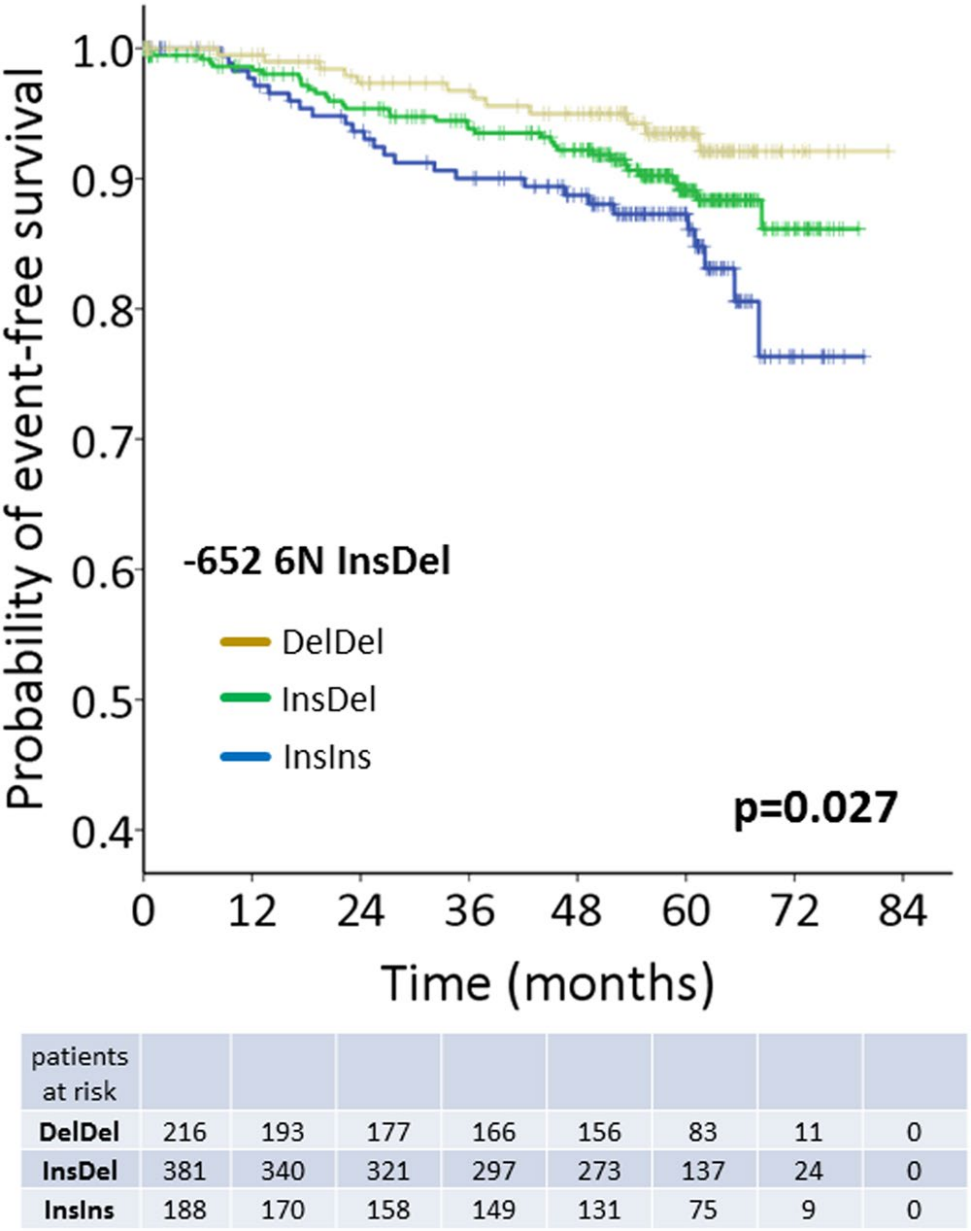
Prognostic relevance of CASP8 -652InsDel in primary breast cancer. Kaplan-Meier curves comparing disease-free survival (DFS) probability in patients with a *CASP8* -652 InsIns, -652InsDel and -652DelDel genotypes. Indicated p-value was calculated by log-rank test for trend.

Kaplan-Meier analysis revealed an allele-dose dependent influence of *CASP8* -652 InsDel on DFS (Fig. 1, p = 0.027) and, according to univariate analysis, homozygous deletion carriers had the lowest risk of death (HR = 0.41; 95% CI = 0.206–0.807; p = 0.010, Supplementary Table 2). Subsequently, we performed multivariate Cox-regression analysis, adjusted for established risk factors in breast cancer (age, tumor stage, nodal status, grading, tumor type, HR-status and HER2-status), and revealed that the -652DelDel variant was an independent prognostic factor for improved DFS (HR = 0.36; 95% CI = 0.174–0.726; p = 0.005; Supplementary Table 3). HER2-receptor status itself was not an independent unfavorable prognostic factor among multivariate Cox-regression analysis (p = 0.766), which might be confounded by trastuzumab therapy in all patients with HER2-positivity. Therefore, although the -652InsDel polymorphism correlated with HER2-receptor status, we can exclude that the prognostic relevance of the -652Del allele is collinear to HER2-receptor status.

In conclusion, we show that the *CASP8* -652 deletion is an allele-dose dependent prognostically favorable factor for DFS in breast cancer and that homozygosity for the -652Del variant is an independent predictor for improved DFS.

### Prognostic relevance of *CASP8* Asp302His for breast cancer

**Figure 2:**
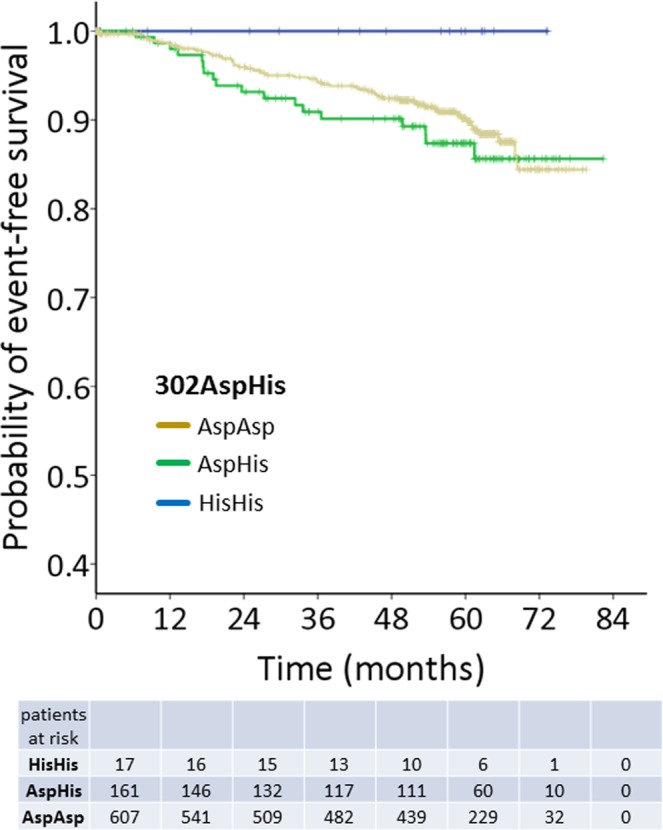
Prognostic relevance of CASP8 Asp302His in early breast cancer. Kaplan-Meier curves comparing disease-free survival (DFS) probability in patients with a *CASP8* 302AspAsp, 302AspHis and 302HisHis genotypes. Since there were no events of recurrence among patients with the 302HisHis genotype during the entire observation time, statistical calculation could not be applied.

We observed that 607/785 patients (77.3%) had an AspAsp genotype, 161/785 patients (20.5%) were heterozygous (AspHis) and 17/785 patients (2.2%) exhibited the rare HisHis genotype (Supplementary Table 4). No significant deviation from Hardy-Weinberg equilibrium was detectable (p = 0.109) and the observed genotype distribution as well as the allelic frequencies (f_His_ = 0.124) were comparable to those previously reported in cancer cases and healthy controls of European ancestry^22^. There was no correlation between the *CASP8* Asp302His polymorphism and the patient’s clinicopathological characteristics, including the intrinsic breast cancer subtypes (Supplementary Table 4). No difference in DFS was observed between patients with a 302 AspAsp genotype and an AspHis genotype (p = 0.406, Supplementary Table 2). However, in patients with a homozygous 302HisHis variant, there was no event of recurrence during the entire observation with a median of 56.4 months (range: 0–82.4 months, Fig. 2).

To sum up, we suggest a prognostically favorable role of the 302HisHis variant for DFS, since we did not observe recurrence of these patients within our observation time. Due to missing events in the HisHis patient group, statistical tests are non-informative in this context.

### Combined analysis of *CASP8* Asp302His and *CASP8* -652 6N InsDel and its prognostic relevance for breast cancer

We used Haploview to analyze a potential linkage of the investigated polymorphisms. We identified four different haplotypes: Del/Asp (f_Del/Asp_ = 0.420), Ins/Asp (f_Ins/Asp_ = 0.456) Del/His (f_Del/His_ = 0.098) and Ins/His (f_Ins/His_ = 0.026). Since this analysis showed that *CASP8* -652 6N InsDel and *CASP8* Asp302His are in linkage disequilibrium to each other (D’ = 0.565 CI = 0.41–0.69), but showed a low correlation (r^2^ = 0.042), we inquired, whether the effects of these two polymorphisms on DFS were independent from each other. Therefore, DFS was individually regressed by (i) both polymorphisms (bivariate analysis) and (ii) by both polymorphisms in addition to established risk factor for breast cancer (tumor stage, nodal status, grading, tumor type, receptor status; multivariable analysis). Interestingly, the -652 DelDel genotype was an independent predictor for improved DFS, according to bivariate (HR = 0.38, 95% CI: 0.190–0.769; p = 0.007, Supplementary Table 2) and multivariate regression analysis (HR = 0.29, 95% CI: 0.136–0.635; p = 0.002, Supplementary Table 5).

**Figure 3:**
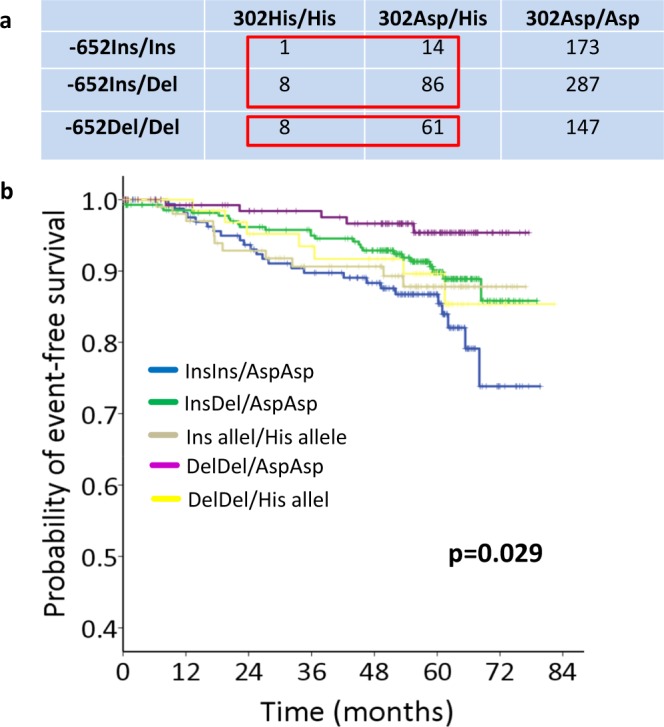
Prognostic relevance of combined CASP8 -652InsDel and Asp302His genotypes in early breast cancer. Seventeen patients belonged to rare diplotypes and needed to be analyzed together with other patients. (**a**) The figure shows how we joined these rare diplotype carriers with the common ones. (**b**) Kaplan-Meier curves comparing disease-free survival (DFS) probability of breast cancer patients with regard to the diplotypes of the -652 6N InsDel and Asp302His polymorphisms.

Moreover, we investigated prognostic significance of combined -652 InsDel and Asp302His genotypes in breast cancer patient and referred to the different *CASP8* diplotypes. Theoretically, 4 haplotypes, according to these polymorphisms, result in 10 diplotypes. However, due to the underlying haplotype frequencies and the given linkage of these polymorphisms, only 5 common diplotypes were detected. Seventeen patients were associated with rare diplotypes and were therefore analyzed, together. Figure 3a shows how we combined rare diplotype carriers with common diplotype carriers. Kaplan-Meier analysis was performed, in order to determine prognostic relevance of *CASP8* diplotypes. We observed an influence of both polymorphisms on DFS (p = 0.029, Fig. 3b).

We conclude that 652InsDel and Asp302His jointly modulate the prognosis of breast cancer.

### Correlation of *CASP8* -652 InsDel and Asp302His with tumor infiltrating lymphocytes in breast cancer

**Figure 4:**
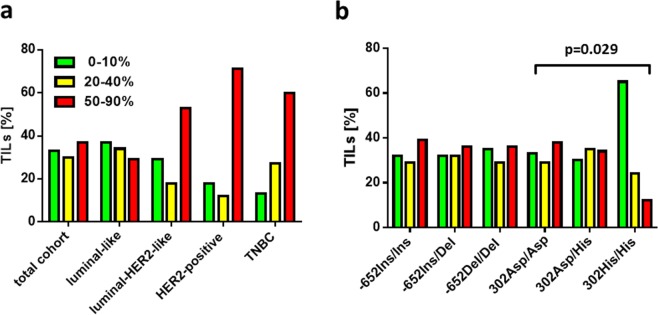
Correlation between stromal TIL concentration and caspase 8 genotypes. (**a**) Bar chart comparing stromal TIL concentration in the entire cohort and among the intrinsic subtypes of breast cancer. (**b**) Bar chart comparing stromal TIL concentration among the caspase 8 genotypes in the pooled patient cohort.

Considering that *CASP8* -652 InsDel and Asp302His have previously been described to promote survival of T-lymphocytes, which suggests a potential immunomodulatory role of these genetic variants^4^, we analyzed intratumoral and stromal TILs among the tumor tissues of all study patients and correlated these findings with the underlying caspase 8 genotypes. Since intratumoral TIL frequency was consistently low (86% of patients had ≤10% intratumoral TILs), we exclusively focused on stromal TILs in our analysis. In the total patient cohort, stromal TIL infiltration was low (≤10%) in 33% of patients, moderate (20–40%) in 30% of patients and high (50–90%) in 37% of patients (Fig. 4a). According to the different intrinsic subtypes of breast cancer, we observed that patients with luminal-like breast cancer had comparatively lower TIL infiltrations (71% low or moderate and only 29% high infiltration), whereas in luminalHER2-like, HER2-positive and TNBC tumors, stromal TILs were more frequent and high infiltration was observed in 53%, 71% and 60%, respectively (Fig. 4a).

We interrogated whether there is an association between TIL concentration and caspase 8 genotypes. For the -652InsDel polymorphism, TIL concentration was comparable between InsIns, InsDel and DelDel genotypes. Thus, for each genotype, the proportion of patients with low, moderate or high TIL concentration was comparable and ranged from 29% to 39%. There was no significant difference between caspase 8 genotypes and TIL concentration. Interestingly, for *CASP8* Asp302His, the majority of patients with the homozygous HisHis genotype (65%) had low TIL concentration and moderate or high TIL concentration was observed in only 24% and 12% of 302His/His patients, respectively (p = 0.029, Fig. 4b). Thus, according to an analysis of all patients, there was a very clear enrichment of the 302HisHis variant in patients with low TIL concentration (65%; p = 0.029).

Subsequently, we inquired, whether the above mentioned association of caspase 8 genotypes with TIL concentration in the total patient cohort can also be found in different subtypes of breast cancer. For luminal HER2-like, non-luminal HER2-positive and TNBC, the total number of patients with a 302HisHis genotype was too low (n ≤ 3) in order to draw any conclusion on this. In the luminal subtype, which was the major subtype in our cohort (76%), there were a total of 12 patients with the 302HisHis genotype. Here again, we observed the clear trend that most of the patients with the 302HisHis genotype (n = 7, 58%) had a low TIL concentration. However, due to the limited number of patients in this group, this trend did not yet reach statistical significance (p = 0.368).

Conclusively, we report that *CASP8* 302 HisHis variant is significantly associated with a tumor phenotype of low immune infiltration in the pooled patient cohort and among luminal breast cancer patients alone (by trend).

## Discussion

In the present study, we observed a favorable influence of the *CASP8* -652Del and the 302HisHis genotype on DFS in early breast cancer and reported that the 302HisHis genotype correlates with low TIL concentration.

As a key apoptosis-regulating gene, caspase 8 is central in initiating apoptosis upon receipt of apoptotic signals from death receptors^23^. A previous study proposed that the *CASP8* -652Del allele, resulting in lower caspase 8 expression, promotes survival of T-lymphocytes after stimulation with cancer cell antigens^4^. Considering that T-lymphocytes are involved in the immune response to malignant cells, the -652Del variant was consequently shown to be associated with less susceptibility for variety of cancer types^4,5,24–26^. However, in patients with a -652InsDel or DelDel genotype, the Del allele is not only present in the genome of T-lymphocytes in the blood but also among pre-malignant cells at the time of tumor onset or among fully malignant tumor cell in an already invasive tumor. Since decreased apoptotic capacity is one of the “*hallmarks of cancer*”^27^, one could assume, that the -652Del allele, which generally lowers caspase 8 mediated apoptosis, could be, for the tumor cells themselves, rather a tumor-promoting than a protective factor. However, a generally lowered caspase 8 mediated apoptotic threshold does not seem to be tumor promoting in existing breast cancer, since we clearly show that the -652Del allele in patients with already existing breast cancer is prognostically favorable. This could be explained by the occurrence of reactive T-lymphocytes with prolonged survival, which counteract immune-escape mechanisms and may confer a more efficient anti-tumor response, resulting in improved DFS. Interestingly, this finding is in agreement with a recent study on gastric cancer, proposing the -652Del allele as favorable prognostic biomarker^28^.

For Asp302His, which is located in the coding region of caspase 8 and which has also been associated with decreased breast cancer susceptibility^4^, we observed that patients with a 302HisHis genotype did not have any event of recurrence during the entire observation time, suggesting also a prognostically favorable influence of the 302HisHis genotype. However, instructive data on the functionality of this polymorphism are still missing and it can only be hypothesized that the effect of a homozygous 302His allele is likewise due to impaired caspase 8 function and decreased apoptosis in T-lymphocytes, possibly by downregulation of caspase 8 auto processing capacity or by downregulation of the catalytic activity of caspase 8^7^. Collectively, our findings complement previous epidemiological findings, associating *CASP8* -652InsDel and *CASP8* Asp302His with decreased breast cancer susceptibility^4,5,24–26^ and extend clinical relevance of the -652Del variant from predicting breast cancer susceptibility to prognostic stratification.

In a previous pilot study, we have already investigated prognostic influence of -652 InsDel and Asp302His in breast cancer patients with regard to OS^29^. We found that, in patients with existing breast cancer, both, the -652Del and the 302His variant were unfavorable prognostic factors, indicating shorter OS. However, this previous investigation was performed in a historic patient cohort (primary diagnoses between 1989 and 1993) with only a limited number of patients (n = 200), which were not treated according to current clinical standard of care. In spite of a distinct risk profile of this historic patient cohort, the majority of patients (56%) did not receive any adjuvant treatment. In addition, although 68% of patients were ER-positive, only 44% of patients received an adjuvant therapy with tamoxifen (and/or CMF)^29^. Furthermore, although HER2-status was available in the majority of patients, a HER2-directed therapy with trastuzumab was not yet approved for clinical use at that time. Therefore, our previous findings have to be interpreted with care. In our current investigation, we can now draw a statistically substantiated conclusion on 785 early breast cancer patients, who were treated according to the current state of care as part of the multicenter prospective PiA study (NCT01592825) and we conclude that both polymorphism have a favorable influence on DFS. Considering that 302 AspHis is a very rare polymorphism and that the number of death events was too small during our observation time, we could not include OS into our survival analysis.

Considering that -652 InsDel and Asp302His could have an influence on tumor surveillance and the anti-tumor response of T-lymphocytes, we for the first time correlated caspase 8 genotypes with the concentration of TILs. Since intratumoral TIL concentration was consistently low, we focused only on stromal TILs. This was considered reasonable, since TILs are usually more abundant in stromal compared to intratumoral compartments and the assessment of stromal TILs has emerged as the most reproducible immune parameter scored by pathologists^10^. In our patients, luminal-like tumors, represented by almost 75% of the patients, had the lowest concentration of TILs, whereas luminal HER2-like tumors and TNBC showed higher TIL concentrations. This finding was not surprising, since especially TNBC, due to its genetic instability and its high mutation rate, is known to represent the breast cancer subtype with the highest lymphocyte infiltrate, indicating that these tumors are more immunogenic compared to luminal tumors^10,30^. Since breast cancer is generally supposed to be an immunogenic tumor, clinical relevance of TIL concentration has been intensively studied. Increased TIL concentration is known to predict response to neoadjuvant chemotherapy, independently from breast cancer subtype. Moreover, increased TIL concentration is associated with a survival benefit in HER2-positive breast cancer and TNBC^12^.

We observed that patients with a homozygous 302 HisHis genotype are more likely to have a low TIL concentration. This association was evident among the analysis of the entire cohort and among patients with luminal-like HER2 negative tumors (by trend), which represent the majority of patients. In luminal breast cancer, low rather than high TIL concentration was reported to indicate improved survival, suggesting a different biology of immunoinfiltration in this subtype^12^. Moreover, it has been speculated that increased TILs in ER-positive breast cancer are associated with more aggressive tumor biology and endocrine resistance^12,31^. In this context, we suggest for the first time a link between a (prognostically favorable) caspase 8 genotype (302 HisHis) and a prognostically favorable condition of immune infiltration (i.e. low TIL-concentration)^12^ and it could be hypothesized that the HisHis genotype pre-defines response to endocrine therapy. In our study, there was no event of recurrence among all ER-positive breast cancer patients with the HisHis genotype (observation time around 4 to 5 years), suggesting that there was also no event of primary endocrine resistance in this group. However, since the His variant is extremely rare, the number of 302HisHis patients was too small in order to draw a statistically substantiated conclusion.

We did not observe any correlation between the -652Del variant and the concentration of TILs, neither among the entire cohort nor among the different breast cancer subtypes. Therefore, it seems that caspase 8, although it promotes survival of T-lymphocytes, does not influence the overall count of TILs. Considering the complex composition of breast cancer immunoinfiltrates (including CD8^+^ and CD4^+^ T-lymphocytes, regulatory T-cells or macrophages), future studies will have to show whether the -652 InsDel polymorphism takes an influence on TIL composition.

The strength of our study is that our results are based on a large patient cohort with a long observation time. We propose a prognostically favorable role of the *CASP8* -652Del and the 302HisHis genotype in primary breast cancer patients and suggest for the first time an association between a polymorphism in an apoptosis-related gene and the immunophenotype of breast cancer. As a limitation of our study, we did not perform subtyping of TILs and did not include the effects of therapy into our analysis. Moreover, since especially Asp302His is a very rare genotype, the number of patients with a HisHis variant was limited.

## Conclusions

We report on a prognostically favorable influence of the *CASP8* -652Del and the 302HisHis in early breast cancer and demonstrate that the 302HisHis genotype correlates with low TIL concentration. Our findings add a new layer of complexity to immunological interaction in breast cancer and indicate that the immunophenotype of a given tumor could pre-defined by genetic variability in apoptosis regulating genes. This could be in addition to known factors affecting the immunophenotype, such as the rate of somatic mutations or the degree of neo-antigenicity^32^. Our findings strongly encourage further investigation of caspase polymorphisms as prognostic biomarkers or as response-predictors for endocrine therapy or immunotherapy.

## Supplementary information

Supplementary Tables
