# Supplementary material for: Association of caspase 8 polymorphisms -652 6N InsDel and Asp302His with progression-free survival and tumor infiltrating lymphocytes in early breast cancer
Source: Sci Rep. 2019 Aug 29;9:12594. doi: 10.1038/s41598-019-47601-x (PMC6715668; doi:10.1038/s41598-019-47601-x)
Supplement: Supplementary file 1 — Supplementary Tables [file 41598_2019_47601_MOESM1_ESM.docx]

***Association of caspase 8 polymorphisms -652 6N InsDel and Asp302His with progression-free survival and tumor infiltrating lymphocytes in primary breast cancer***

Jan Dominik Kuhlmann, Hagen Sjard Bachmann, Theresa Link, Pauline Wimberger, Eric Kröber, Christoph Thomssen, Brahima Mallé, Martina Vetter, Eva Kantelhardt

Supplementary Tables

**Tables**

| **Table 1: Clinicopathological characteristics at primary diagnosis and *CASP8* -652 6N InsDel genotype distribution** | | | | | | | | | |
| --- | --- | --- | --- | --- | --- | --- | --- | --- | --- |
|  |  | | **CASP8 -652 6N InsDel genotype** | | | | | | |
|  |  | | **InsIns** | | **InsDel** | | **DelDel** |  | |
| **Characteristic** | **Number** | **%** | **n** | **%** | **n** | **%** | **n** | **%** | ***P-value** |
| **Total** | 785 |  | 188 | 23.9 | 381 | 48.5 | 216 | 27.5 |  |
| **Age** |  |  |  |  |  |  |  |  |  |
| <35 years | 15 | 1.9 | 5 | 2.7 | 6 | 1.6 | 4 | 1.9 |  |
| 35-50 years | 176 | 22.4 | 50 | 26.6 | 80 | 21.0 | 46 | 21.3 |  |
| >50 years | 594 | 75.7 | 133 | 70.7 | 295 | 77.4 | 166 | 76.9 | 0.477 |
| **Mean Age (Min – Max)** | 62.3 | (22 - 90) | 61.9 | (28 - 88) | 62.6 | (22 - 90) | 62.0 | (31 - 88) |  |
| **Menopausal state** |  |  |  |  |  |  |  |  |  |
| postmenopausal | 567 | 72.2 | 124 | 66.0 | 284 | 74.5 | 159 | 73.6 |  |
| premenopausal | 162 | 20.6 | 45 | 23.9 | 72 | 18.9 | 45 | 20.8 |  |
| premenopausal >50 | 3 | 0.4 | 1 | 0.5 | 1 | 0.3 | 1 | 0.5 |  |
| perimenopausal | 53 | 6.8 | 18 | 9.6 | 24 | 6.3 | 11 | 5.1 | 0.378 |
| **Tumorsize Median in cm (Min - Max)** | 2.0 | (0.2 - 11.0) | 2.0 | (0.2 - 10.0) | 2 | (0.3 - 11.0) | 1.9 | (0.3 - 9.0) |  |
| **Tumor stage** |  |  |  |  |  |  |  |  |  |
| pT1 | 400 | 51 | 94 | 50 | 191 | 50.1 | 115 | 53.2 |  |
| pT2 | 342 | 43.6 | 80 | 42.6 | 171 | 44.9 | 91 | 42.1 |  |
| pT3 | 36 | 4.6 | 13 | 6.9 | 15 | 3.9 | 8 | 3.7 |  |
| pT4 | 7 | 0.9 | 1 | 0.5 | 4 | 1 | 2 | 0.9 | 0.687 |
| **Nodal status** |  |  |  |  |  |  |  |  |  |
| N0 | 480 | 61.1 | 117 | 62.2 | 231 | 60.6 | 132 | 61.1 |  |
| N1 | 223 | 28.4 | 51 | 27.1 | 114 | 29.9 | 58 | 26.9 |  |
| N2 | 51 | 6.5 | 12 | 6.4 | 24 | 6.3 | 15 | 6.9 |  |
| N3 | 31 | 3.9 | 8 | 4.3 | 12 | 3.1 | 11 | 5.1 | 0.908 |
| **Histology** |  |  |  |  |  |  |  |  |  |
| ductal | 627 | 79.9 | 151 | 80.3 | 303 | 79.5 | 173 | 80.1 |  |
| lobular | 115 | 14.6 | 25 | 13.3 | 59 | 15.5 | 31 | 14.4 |  |
| other | 43 | 5.5 | 12 | 6.4 | 19 | 5.0 | 12 | 5.6 | 0.926 |
| **Grading** |  |  |  |  |  |  |  |  |  |
| G1 | 89 | 11.3 | 27 | 14.4 | 47 | 12.3 | 15 | 6.9 |  |
| G2 | 489 | 62.3 | 108 | 57.4 | 239 | 62.7 | 142 | 65.7 |  |
| G3 | 207 | 26.4 | 53 | 28.2 | 95 | 24.9 | 59 | 27.3 | 0.122 |
| **Estrogen receptor status** |  |  |  |  |  |  |  |  |  |
| negative | 121 | 15.4 | 28 | 14.9 | 56 | 14.7 | 37 | 17.1 |  |
| positive | 664 | 84.6 | 160 | 85.1 | 325 | 85.3 | 179 | 82.9 | 0.713 |
| **Progesteron receptor status** |  |  |  |  |  |  |  |  |  |
| negative | 234 | 29.8 | 46 | 24.5 | 124 | 32.5 | 64 | 29.6 |  |
| positive | 551 | 70.2 | 142 | 75.5 | 257 | 67.5 | 152 | 70.4 | 0.140 |
| **Hormone receptor status** |  |  |  |  |  |  |  |  |  |
| negative | 114 | 14.5 | 27 | 14.4 | 54 | 14.2 | 33 | 15.3 |  |
| positive | 671 | 85.5 | 161 | 85.6 | 327 | 85.8 | 183 | 84.7 | 0.923 |
| **HER2 status** |  |  |  |  |  |  |  |  |  |
| negative | 675 | 86.0 | 165 | 87.8 | 336 | 88.2 | 174 | 80.6 |  |
| positive | 110 | 14.0 | 23 | 12.2 | 45 | 11.8 | 42 | 19.4 | **0.026** |
| **Breast cancer subtype** |  |  |  |  |  |  |  |  |  |
| luminal-like | 596 | 75.9 | 143 | 76.1 | 300 | 78.7 | 153 | 70.8 |  |
| luminalHER2-like | 75 | 9.6 | 18 | 9.6 | 27 | 7.1 | 30 | 13.9 |  |
| HER2 | 35 | 4.5 | 5 | 2.7 | 18 | 4.7 | 12 | 5.6 |  |
| TNBC | 79 | 10.1 | 22 | 11.7 | 36 | 9.4 | 21 | 9.7 | 0.105 |
| **Median Follow-Up (months)** | 56.4 |  |  |  |  |  |  |  |  |

**P-values were calculated using the Pearson’s Chi² test for categorical data.*

**Table 2: Prognostic relevance of *CASP8* -652 6N and *CASP8* Asp302His polymorphism (univariate and bivariate Cox-regression analysis)**

| **Table 2**  **Variable** | **Hazard ratio** | **95% CI** | ***P*** |
| --- | --- | --- | --- |
| **Univariate Analysis** |  |  |  |
| **-652 6N Del** |  |  |  |
| InsIns | 1 |  |  |
| InsDel | 0.664 | 0.400-1.104 | 0.114 |
| DelDel | 0.407 | 0.206-0.807 | **0.010** |
|  |  |  |  |
| **Asp302His** |  |  |  |
| AspAsp | 1 |  |  |
| AspHis | 1.253 | 0.736-2.134 | 0.406 |
| HisHis |  | **no event of recurrence** |  |
|  |  |  |  |
| **Bivariate Analysis** |  |  |  |
| -**652 6N Del** |  |  |  |
| InsIns | 1 |  |  |
| InsDel | 0.635 | 0.379-1.063 | 0.084 |
| DelDel | 0.382 | 0.190-0.769 | **0.007** |
| Asp302His |  |  |  |
| AspAsp | 1 |  |  |
| AspHis | 1.483 | 0.858-2.564 | 0.158 |
| HisHis |  | **no event of recurrence** |  |

| **Table 3: Prognostic relevance of *CASP8* -652 6N polymorphism (multivariate Cox-regression analysis)** | | | |
| --- | --- | --- | --- |
| **Characteristic** | **Adjusted hazard ratio** | **95 CI** | **P** |
| **Age** | 1.006 | 0.989 – 1.024 | 0.463 |
| **Tumor stage (Reference: pT1; n=400)** |  |  |  |
| pT2 n=342 | 1.827 | 1.058 – 3.156 | **0.031** |
| pT3 n=36 | 2.881 | 1.140 – 7.285 | **0.025** |
| pT4 n=7 | 0.653 | 0.076 – 5.025 | 0.653 |
| **Nodal status (Reference: N0; n=480)** |  |  |  |
| N1 n=223 | 1.265 | 0.715 – 2.238 | 0.419 |
| N2 n=51 | 2.940 | 1.413 – 6.119 | **0.004** |
| N3 n=31 | 6.304 | 2.988 – 13.298 | **0.000** |
| **Grading (Reference: G1;** **n=89)** |  |  |  |
| G2 n=489 | 6.538 | 0.889 – 48.102 | 0.065 |
| G3 n=207 | 7.615 | 1.007 – 57.600 | **0.049** |
| **Tumor type (Reference: Ductal; n=627)** |  |  |  |
| lobular n=115 | 0.310 | 0.119 – 0.804 | **0.016** |
| others n=43 | 0.537 | 0.163 – 1.772 | 0.308 |
| **HR status (Reference: positive; n=671)** |  |  |  |
| negative n=114 | 1.957 | 1.113 – 3.443 | **0.020** |
| **HER2 status (Reference: negative; n=675)** |  |  |  |
| positive n=110 | 1.096 | 0.599 – 2.007 | 0.766 |
| **-652 6N InsDel (Reference: InsIns; n=188)** |  |  |  |
| InsDel n=381 | 0.709 | 0.417 – 1.205 | 0.203 |
| DelDel n=216 | 0.355 | 0.174 – 0.726 | **0.005** |

| **Table 4: Clinicopathological characteristics at primary diagnosis and *CASP8* Asp302His genotype distribution** | | | | | | | | | |
| --- | --- | --- | --- | --- | --- | --- | --- | --- | --- |
|  |  | | **CASP8 Asp302His genotype** | | | | | | |
|  |  | | **HisHis** | | **AspHis** | | **AspAsp** |  | |
| **Characteristic** | **Number** |  | **n** | % | **n** | % | **n** | % | ****P*-value** |
| **Total** | 785 |  | 17 | 2.2 | 161 | 20.5 | 607 | 77.3 |  |
| **Age** |  |  |  |  |  |  |  |  |  |
| <35 years | 15 | 1.9 | 0 | 0.0 | 6 | 3.7 | 9 | 1.5 |  |
| 35-50 years | 176 | 22.4 | 1 | 5.9 | 32 | 19.9 | 143 | 23.6 |  |
| >50 years | 594 | 75.7 | 16 | 94.1 | 123 | 76.4 | 455 | 75.0 | 0.117 |
| **Mean Age (Min – Max)** | 62.3 | (22 - 90) | 65.24 | (43 - 80) | 62.3 | (22 - 89) | 62.2 | (28 - 90) |  |
| **Menopausal state** |  |  |  |  |  |  |  |  |  |
| postmenopausal | 567 | 72.2 | 16 | 94.1 | 119 | 73.9 | 432 | 71.2 |  |
| premenopausal | 162 | 20.6 | 1 | 5.9 | 33 | 20.5 | 128 | 21.1 |  |
| premenopausal >50 | 3 | 0.4 | 0 | 0 | 1 | 0.6 | 2 | 0.3 |  |
| perimenopausal | 53 | 6.8 | 0 | 0 | 8 | 5.0 | 45 | 7.4 | 0.447 |
| **Tumorsize Median in cm (Min - Max)** | 2.0 | (0.2 - 11.0) | 1.8 | (0.8 - 7.0) | 2.1 | (0.3 - 11.0) | 2.0 | (0.2 - 11.0) |  |
| **Tumor stage** |  |  |  |  |  |  |  |  |  |
| pT1 | 400 | 51 | 11 | 64.7 | 78 | 48.4 | 311 | 51.2 |  |
| pT2 | 342 | 43.6 | 5 | 29.4 | 69 | 42.9 | 268 | 44.2 |  |
| pT3 | 36 | 4.6 | 1 | 5.9 | 10 | 6.2 | 25 | 4.1 |  |
| pT4 | 7 | 0.9 | 0 | 0 | 4 | 2.5 | 3 | 0.5 | 0.187 |
| **Nodal status** |  |  |  |  |  |  |  |  |  |
| N0 | 480 | 61.1 | 13 | 76.5 | 94 | 58.4 | 373 | 61.4 |  |
| N1 | 223 | 28.4 | 2 | 11.8 | 44 | 27.3 | 177 | 29.2 |  |
| N2 | 51 | 6.5 | 2 | 11.8 | 14 | 8.7 | 35 | 5.8 |  |
| N3 | 31 | 3.9 | 0 | 0 | 9 | 5.6 | 22 | 3.6 | 0.315 |
| **Histology** |  |  |  |  |  |  |  |  |  |
| ductal | 627 | 79.9 | 12 | 70.6 | 127 | 78.9 | 488 | 80.4 |  |
| lobular | 115 | 14.6 | 4 | 23.5 | 21 | 13.0 | 90 | 14.8 |  |
| other | 43 | 5.5 | 1 | 5.9 | 13 | 8.1 | 29 | 4.8 | 0.411 |
| **Grading** |  |  |  |  |  |  |  |  |  |
| G1 | 89 | 11.3 | 1 | 5.9 | 22 | 13.7 | 66 | 10.9 |  |
| G2 | 489 | 62.3 | 15 | 88.2 | 103 | 64.0 | 371 | 61.1 |  |
| G3 | 207 | 26.4 | 1 | 5.9 | 36 | 22.4 | 170 | 28.0 | 0.104 |
| **Estrogen receptor status** |  |  |  |  |  |  |  |  |  |
| negative | 121 | 15.4 | 2 | 11.8 | 29 | 18.0 | 90 | 14.8 |  |
| positive | 664 | 84.6 | 15 | 88.2 | 132 | 82.0 | 517 | 85.2 | 0.558 |
| **Progesteron receptor status** |  |  |  |  |  |  |  |  |  |
| negative | 234 | 29.8 | 7 | 41.2 | 46 | 28.6 | 181 | 29.8 |  |
| positive | 551 | 70.2 | 10 | 58.8 | 115 | 71.4 | 426 | 70.2 | 0.558 |
| **Hormone receptor status** |  |  |  |  |  |  |  |  |  |
| negative | 114 | 14.5 | 2 | 11.8 | 27 | 16.8 | 85 | 14.0 |  |
| positive | 671 | 85.5 | 15 | 88.2 | 134 | 83.2 | 522 | 86.0 | 0.640 |
| **HER2 status** |  |  |  |  |  |  |  |  |  |
| negative | 675 | 86.0 | 13 | 76.5 | 136 | 84.5 | 526 | 86.7 |  |
| positive | 110 | 14.0 | 4 | 23.5 | 25 | 15.5 | 81 | 13.3 | 0.405 |
| **Breast cancer subtype** |  |  |  |  |  |  |  |  |  |
| luminal-like | 596 | 75.9 | 12 | 70.6 | 117 | 72.7 | 467 | 76.9 |  |
| luminalHER2-like | 75 | 9.6 | 3 | 17.6 | 17 | 10.6 | 55 | 9.1 |  |
| HER2 | 35 | 4.5 | 1 | 5.9 | 8 | 5.0 | 26 | 4.3 |  |
| TNBC | 79 | 10.1 | 1 | 5.9 | 19 | 11.8 | 59 | 9.7 | 0.818 |

**P-values were calculated using the Pearson’s Chi² test for categorical data.*

| **Table 5: Prognostic relevance of *CASP8* -652 6N and *CASP8* Asp302His polymorphism (combined multivariate Cox-regression analysis)** | | | |
| --- | --- | --- | --- |
| **Characteristic** | **Adjusted  hazard ratio** | **95% CI** | **P** |
| **Age** | 1.007 | 0.990 - 1.025 | 0.394 |
| **Tumor stage (Reference: pT1; n=400)** |  |  |  |
| pT2 n=342 | 1.812 | 1.046 – 3.136 | **0.034** |
| pT3 n=36 | 2.859 | 1.134 – 7.210 | **0.026** |
| pT4 n=7 | 0.532 | 0.065 – 4.380 | 0.558 |
| **Nodal status (Reference: N0; n=480)** |  |  |  |
| N1 n=223 | 1.262 | 0.714 – 2.232 | 0.423 |
| N2 n=51 | 2.987 | 1.429 – 6.245 | **0.004** |
| N3 n=31 | 6.161 | 2.900 – 13.091 | **0.000** |
| **Grading (Reference: G1;** **n=89)** |  |  |  |
| G2 n=489 | 7.226 | 0.980 – 53.296 | 0.052 |
| G3 n=207 | 8.545 | 1.124 – 64.941 | **0.038** |
| **Tumor type (Reference: Ductal;** **n=627)** |  |  |  |
| Lobular n=115 | 0.316 | 0.121 – 0.820 | **0.018** |
| others n=43 | 0.499 | 0.151 – 1.655 | 0.256 |
| **HR status (Reference: positive;**  **n=671)** |  |  |  |
| negative n=114 | 1.831 | 1.032 – 3.248 | **0.039** |
| **HER2 status (Reference: negative;**  **n=675)** |  |  |  |
| positive n=110 | 1.084 | 0.591 – 1.990 | 0.794 |
| **Asp302His (Reference: AspAsp;**  **n=607)** |  |  |  |
| AspHis n=161 | 1.715 | 0.953 – 3.087 | 0.072 |
| HisHis n=17 | **no event of recurrence** |  |  |
| -**652 6N InsDel (Reference: InsIns;**  **n=188)** |  |  |  |
| InsDel n=381 | 0.652 | 0.379 – 1.122 | 0.123 |
| DelDel n=216 | 0.294 | 0.136 – 0.635 | **0.002** |
